# Season of detection of breast cancer.

**DOI:** 10.1038/bjc.1991.360

**Published:** 1991-09

**Authors:** B. Mason, I. Holdaway


*Br. J. Cancer* (1991), **64**, 609 © Macmillan Press Ltd., 1991

**LETTER TO THE EDITOR**

### Season of detection of breast cancer

**Sir** – In a recent letter Galea and Blamey (*Br. J. Cancer* (1991), **63**, 157) have been unable to find a difference in either frequency of tumour detection or patient survival associated with season of detection of breast cancer. This is in contrast to our recent publication 'Season of initial discovery of tumour as an independent variable predicting survival in breast cancer', *Br. J. Cancer* (1990) **61**, 137–141.

It is abundantly desirable that this possible seasonal phenomenon is sought for in breast cancer data files from various geographic regions. It is, of course, important that such analyses involve the month of first detection of tumour by the patient rather than simply the month of surgery or biopsy, since the time between these events is variable.

We acknowledged in our paper the independence and power of the known prognostic variables in breast cancer, particularly nodal status, tumour size, and steroid hormone receptor status. Using proportional hazards regression analysis the season of tumour detection remained a significant prognostic variable when allowing for these factors, although statistically weaker than the others. It is therefore unlikely to exert a major influence on the results of treatment or prognosis if allowance is made for the major prognostic variables.

An increase in breast tumour detection spring/summer is now documented in seven published papers. We do acknowledge however that there may be geographic regions where the seasonal detection of tumours has not been apparent and these findings may not have been published. If there is no difference in season of tumour detection with regards to incidence then it is unlikely that season of tumour detection would influence survival in that particular group of women. Galea and Blamey were not able to show that women $>= 50$ years with oestrogen receptors in the tumour had any difference in survival according to season of tumour detection. It would be of interest to know whether they were able to show a survival difference according to presence of oestrogen receptors in this group irrespective of season. It is noteworthy that our study included all cases over 9 years from one geographic region, whereas it is unclear whether the Nottingham data is a record of all cases of breast cancer in that area. There was also no screening by mammography during the 9 years of the collection of the Auckland data file and this may be a further point of difference between the patient groups. We agree with Galea and Blamey that further reports will be necessary before this debate can be resolved.

yours etc,

Barbara Mason (Scientific Officer),
Ian Holdaway (Assoc Professor of Endocrinology),
Auckland Breast Cancer Study Group,
University of Auckland,
Auckland, New Zealand.


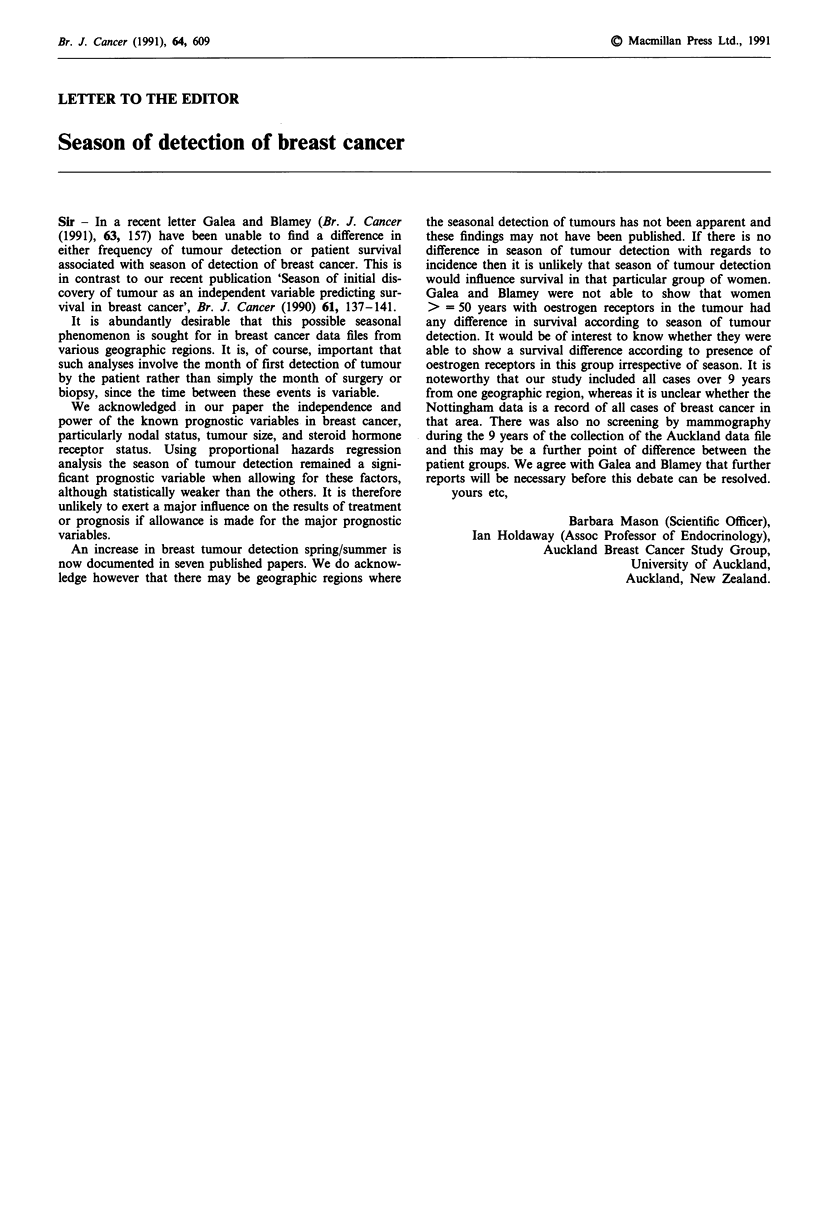


## References

[OCR_00009] Galea M. H., Blamey R. W. (1991). Season of initial detection in breast cancer.. Br J Cancer.

